# 1,1′-Binaphthyl-2,2′-diyl benzyl­phos­phoramidate

**DOI:** 10.1107/S1600536811046861

**Published:** 2011-11-16

**Authors:** Ravikumar R Gowda, Venkatachalam Ramkumar, Debashis Chakraborty

**Affiliations:** a Department of Chemistry, IIT Madras, Chennai, TamilNadu, India

## Abstract

In the title compound, C_27_H_20_NO_3_P, the P atom exhibits a somewhat distorted PNO_3_ tetra­hedral geometry, with the O—P—O angle for the binaphthyl fragment being 102.82 (6)°. The dihedral angle between the naphthyl ring systems is 59.00 (2)°. In the crystal, inversion dimers linked by pairs of N—H⋯O hydrogen bonds generate *R*_2_^2^(8) loops.

## Related literature

For background to organo­phospho­rus chemistry, see: Malik *et al.* (2010 ▶). For related structures, see: Gowda *et al.* (2010*a* ▶,*b* ▶, 2011 ▶).
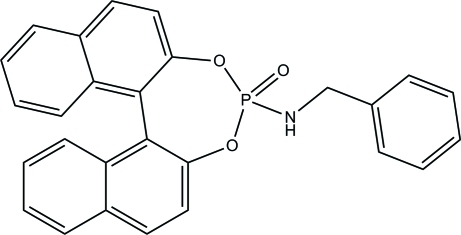


## Experimental

### 

#### Crystal data

C_27_H_20_NO_3_P
*M* *_r_* = 437.41
Monoclinic, 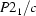

*a* = 13.7998 (4) Å
*b* = 11.0667 (3) Å
*c* = 14.7487 (5) Å
β = 112.674 (1)°
*V* = 2078.31 (11) Å^3^
*Z* = 4
Mo *K*α radiation
μ = 0.16 mm^−1^
*T* = 298 K
0.35 × 0.27 × 0.22 mm

#### Data collection

Bruker APEXII CCD diffractometer
Absorption correction: multi-scan (*SADABS*; Bruker, 2004 ▶) *T*_min_ = 0.945, *T*_max_ = 0.965
29246 measured reflections
5053 independent reflections
3766 reflections with *I* > 2σ(*I*)
*R*_int_ = 0.053

#### Refinement

*R*[*F* ^2^ > 2σ(*F* ^2^)] = 0.039
*wR*(*F* ^2^) = 0.104
*S* = 1.03
5053 reflections
293 parameters
H atoms treated by a mixture of independent and constrained refinement
Δρ_max_ = 0.32 e Å^−3^
Δρ_min_ = −0.51 e Å^−3^

### 

Data collection: *APEX2* (Bruker, 2004 ▶); cell refinement: *SAINT* (Bruker, 2004 ▶); data reduction: *SAINT*; program(s) used to solve structure: *SHELXS97* (Sheldrick, 2008 ▶); program(s) used to refine structure: *SHELXL97* (Sheldrick, 2008 ▶); molecular graphics: *ORTEP-3* (Farrugia, 1997 ▶); software used to prepare material for publication: *SHELXL97*.

## Supplementary Material

Crystal structure: contains datablock(s) global, I. DOI: 10.1107/S1600536811046861/hb6484sup1.cif

Structure factors: contains datablock(s) I. DOI: 10.1107/S1600536811046861/hb6484Isup2.hkl

Supplementary material file. DOI: 10.1107/S1600536811046861/hb6484Isup3.cml

Additional supplementary materials:  crystallographic information; 3D view; checkCIF report

## Figures and Tables

**Table 1:** Hydrogen-bond geometry (Å, °)

*D*—H⋯*A*	*D*—H	H⋯*A*	*D*⋯*A*	*D*—H⋯*A*
N1—H1*N*⋯O3^i^	0.90 (2)	2.01 (2)	2.9015 (17)	170.2 (18)

Symmetry code: (i) 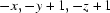.

**Fig. 1.**
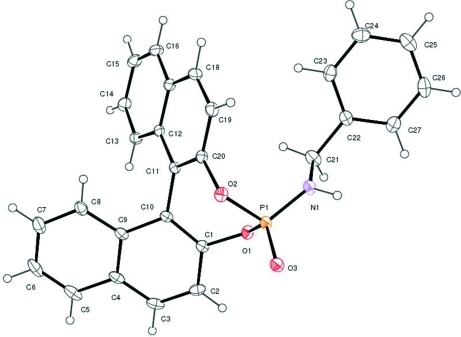
ORTEP of the molecule with atoms represented as 30% probability ellipsoids.

## supplementary crystallographic information

### Comment

In the recent years, we were interested in the chemistry of phosphorus (V) compounds (Malik *et al.*, 2010). One of our major objectives has been the use of such reagents for ring-opening polymerization reactions (Gowda *et al.*, 2011). Recently, we have published two phosphoric acid derivatives (Gowda *et al.*, 2010a,b). The title compound described here is the new benzylphosphoroamidate derived from binol phosphorochloridate and benzyl amine.

The P=O bond length is slightly shorter than the P-O length. All the bond lengths and angles are in agreement with literature precedents (Gowda *et al.*, 2011).

### Experimental

To a stirred solution of binol (200 mg, 0.69 mmol) in CH_2_Cl_2_ (20 mL) under nitrogen atm at 0 °C was added triethylamine (2 ml, 13.96 mmol) dropwise. To the above reaction mixture POCl_3_ (0.07 mL, 0.69 mmol) was added dropwise, formation of HCl was observed. Reaction mixture stirred at 0 °C for 10 mins up to room temperature and continued stirring for 4 h. To the above reaction mixture benzyl amine (0.38 mL, 3.49 mmol)was added at room temperature and the reaction mixture was stirred for 2.5 h. The reaction mixture was evaporated to dryness. Mass was dissoved in CH_2_Cl_2_ (30 ml) and washed with water (5 mL) then with saturated brine (5 ml). Organic layer was dried over Na_2_SO_4_, filtered and evaporated to dryness. This was further purified by column chromatography to yield a colourless solid.

### Refinement

All hydrogen atoms except N atom were fixed geometrically and allowed to ride on the parent carbon atoms with aromatic C-H = 0.93 Å and methylene C-H = 0.96 Å. The displacement parameters were set for phenyl H atoms at *U*_iso_(H) = 1.2*U*_ eq_(C) and for methylene H atoms at *U*_iso_(H) = 1.5*U*_eq_(C).N atom was identified by fourier mapping and were fixed.

### Crystal data

**Table:**

C_27_H_20_NO_3_P	*F*(000) = 912
*M**_r_* = 437.41	*D*_x_ = 1.398 Mg m^−^^3^
Monoclinic, *P*2_1_/*c*	Mo *K*α radiation, λ = 0.71073 Å
Hall symbol: -P 2ybc	Cell parameters from 9423 reflections
*a* = 13.7998 (4) Å	θ = 2.4–28.1°
*b* = 11.0667 (3) Å	µ = 0.16 mm^−^^1^
*c* = 14.7487 (5) Å	*T* = 298 K
β = 112.674 (1)°	Block, colourless
*V* = 2078.31 (11) Å^3^	0.35 × 0.27 × 0.22 mm
*Z* = 4	

### Data collection

**Table:**

Bruker APEXII CCD diffractometer	5053 independent reflections
Radiation source: fine-focus sealed tube	3766 reflections with *I* > 2σ(*I*)
graphite	*R*_int_ = 0.053
phi and ω scans	θ_max_ = 28.3°, θ_min_ = 1.6°
Absorption correction: multi-scan (*SADABS*; Bruker, 2004)	*h* = −18→18
*T*_min_ = 0.945, *T*_max_ = 0.965	*k* = −14→14
29246 measured reflections	*l* = −17→19

### Refinement

**Table:**

Refinement on *F*^2^	Primary atom site location: structure-invariant direct methods
Least-squares matrix: full	Secondary atom site location: difference Fourier map
*R*[*F*^2^ > 2σ(*F*^2^)] = 0.039	Hydrogen site location: inferred from neighbouring sites
*wR*(*F*^2^) = 0.104	H atoms treated by a mixture of independent and constrained refinement
*S* = 1.03	*w* = 1/[σ^2^(*F*_o_^2^) + (0.0445*P*)^2^ + 0.951*P*] where *P* = (*F*_o_^2^ + 2*F*_c_^2^)/3
5053 reflections	(Δ/σ)_max_ = 0.001
293 parameters	Δρ_max_ = 0.32 e Å^−^^3^
0 restraints	Δρ_min_ = −0.51 e Å^−^^3^

### Special details

**Table:**

Geometry. All esds (except the esd in the dihedral angle between two l.s. planes) are estimated using the full covariance matrix. The cell esds are taken into account individually in the estimation of esds in distances, angles and torsion angles; correlations between esds in cell parameters are only used when they are defined by crystal symmetry. An approximate (isotropic) treatment of cell esds is used for estimating esds involving l.s. planes.
Refinement. Refinement of F^2^ against ALL reflections. The weighted R-factor wR and goodness of fit S are based on F^2^, conventional R-factors R are based on F, with F set to zero for negative F^2^. The threshold expression of F^2^ > 2sigma(F^2^) is used only for calculating R-factors(gt) etc. and is not relevant to the choice of reflections for refinement. R-factors based on F^2^ are statistically about twice as large as those based on F, and R- factors based on ALL data will be even larger.

### Fractional atomic coordinates and isotropic or equivalent isotropic displacement parameters (Å^2^)

**Table:**

	*x*	*y*	*z*	*U*_iso_*/*U*_eq_	
C1	0.32299 (11)	0.67344 (13)	0.54067 (11)	0.0203 (3)	
C2	0.40314 (13)	0.62679 (14)	0.62471 (12)	0.0265 (4)	
H2	0.3893	0.6035	0.6791	0.032*	
C3	0.50170 (13)	0.61628 (14)	0.62505 (13)	0.0295 (4)	
H3	0.5561	0.5897	0.6817	0.035*	
C4	0.52293 (12)	0.64488 (13)	0.54141 (13)	0.0260 (4)	
C5	0.62343 (13)	0.62768 (15)	0.53816 (15)	0.0354 (4)	
H5	0.6783	0.5996	0.5938	0.042*	
C6	0.64113 (14)	0.65125 (16)	0.45568 (17)	0.0399 (5)	
H6	0.7078	0.6402	0.4553	0.048*	
C7	0.55859 (14)	0.69235 (15)	0.37079 (16)	0.0363 (4)	
H7	0.5705	0.7067	0.3138	0.044*	
C8	0.46115 (13)	0.71132 (14)	0.37106 (13)	0.0275 (4)	
H8	0.4074	0.7382	0.3141	0.033*	
C9	0.44037 (12)	0.69088 (13)	0.45658 (12)	0.0221 (3)	
C10	0.33940 (11)	0.71257 (13)	0.45942 (11)	0.0192 (3)	
C11	0.25437 (12)	0.77459 (13)	0.37799 (11)	0.0190 (3)	
C12	0.26774 (12)	0.89308 (13)	0.34438 (11)	0.0204 (3)	
C13	0.36253 (13)	0.95968 (14)	0.38600 (12)	0.0236 (3)	
H13	0.4200	0.9252	0.4357	0.028*	
C14	0.37069 (14)	1.07407 (14)	0.35407 (13)	0.0291 (4)	
H14	0.4338	1.1158	0.3817	0.035*	
C15	0.28496 (15)	1.12864 (15)	0.28029 (13)	0.0339 (4)	
H15	0.2910	1.2069	0.2600	0.041*	
C16	0.19313 (15)	1.06775 (16)	0.23828 (13)	0.0318 (4)	
H16	0.1368	1.1046	0.1889	0.038*	
C17	0.18144 (13)	0.94851 (15)	0.26850 (11)	0.0245 (3)	
C18	0.08507 (13)	0.88577 (16)	0.22596 (12)	0.0294 (4)	
H18	0.0297	0.9207	0.1743	0.035*	
C19	0.07260 (12)	0.77502 (16)	0.25975 (12)	0.0269 (4)	
H19	0.0088	0.7345	0.2322	0.032*	
C20	0.15695 (12)	0.72271 (14)	0.33655 (11)	0.0211 (3)	
C21	0.02951 (13)	0.81676 (14)	0.48691 (14)	0.0313 (4)	
H21A	0.0745	0.8546	0.4583	0.038*	
H21B	0.0593	0.8325	0.5570	0.038*	
C22	−0.07845 (12)	0.87219 (13)	0.44285 (12)	0.0246 (3)	
C23	−0.11269 (14)	0.93190 (15)	0.35337 (13)	0.0305 (4)	
H23	−0.0699	0.9341	0.3177	0.037*	
C24	−0.20985 (15)	0.98832 (16)	0.31637 (14)	0.0360 (4)	
H24	−0.2317	1.0285	0.2564	0.043*	
C25	−0.27378 (14)	0.98487 (16)	0.36827 (15)	0.0376 (4)	
H25	−0.3385	1.0238	0.3441	0.045*	
C26	−0.24159 (15)	0.92352 (18)	0.45635 (15)	0.0397 (5)	
H26	−0.2853	0.9195	0.4910	0.048*	
C27	−0.14467 (15)	0.86821 (17)	0.49314 (14)	0.0341 (4)	
H27	−0.1235	0.8275	0.5528	0.041*	
N1	0.02726 (10)	0.68495 (11)	0.47013 (10)	0.0239 (3)	
O1	0.22318 (8)	0.68401 (9)	0.54441 (8)	0.0220 (2)	
O2	0.14012 (8)	0.61001 (9)	0.37169 (8)	0.0229 (2)	
O3	0.12166 (8)	0.47812 (10)	0.49874 (8)	0.0260 (3)	
P1	0.12654 (3)	0.60554 (3)	0.47454 (3)	0.02003 (11)	
H1N	−0.0227 (15)	0.6422 (19)	0.4812 (14)	0.038 (5)*	

### Atomic displacement parameters (Å^2^)

**Table:**

	*U*^11^	*U*^22^	*U*^33^	*U*^12^	*U*^13^	*U*^23^
C1	0.0176 (7)	0.0159 (7)	0.0252 (8)	−0.0039 (6)	0.0057 (6)	−0.0015 (6)
C2	0.0310 (9)	0.0196 (8)	0.0227 (8)	−0.0038 (6)	0.0034 (7)	0.0014 (6)
C3	0.0241 (8)	0.0191 (8)	0.0323 (9)	0.0010 (6)	−0.0035 (7)	0.0011 (7)
C4	0.0186 (8)	0.0136 (7)	0.0391 (10)	−0.0006 (6)	0.0036 (7)	−0.0023 (6)
C5	0.0185 (8)	0.0198 (8)	0.0578 (13)	0.0015 (6)	0.0036 (8)	−0.0051 (8)
C6	0.0227 (9)	0.0240 (9)	0.0775 (15)	0.0011 (7)	0.0242 (10)	−0.0060 (9)
C7	0.0344 (10)	0.0246 (9)	0.0605 (13)	0.0016 (7)	0.0300 (10)	0.0000 (8)
C8	0.0256 (8)	0.0188 (7)	0.0418 (10)	0.0011 (6)	0.0170 (8)	0.0013 (7)
C9	0.0187 (7)	0.0118 (7)	0.0345 (9)	−0.0016 (5)	0.0086 (7)	−0.0006 (6)
C10	0.0159 (7)	0.0132 (6)	0.0254 (8)	−0.0021 (5)	0.0046 (6)	−0.0006 (6)
C11	0.0186 (7)	0.0193 (7)	0.0199 (8)	0.0012 (6)	0.0084 (6)	−0.0009 (6)
C12	0.0232 (8)	0.0202 (7)	0.0206 (8)	0.0030 (6)	0.0117 (6)	0.0005 (6)
C13	0.0252 (8)	0.0191 (7)	0.0270 (8)	0.0017 (6)	0.0107 (7)	0.0007 (6)
C14	0.0363 (10)	0.0205 (8)	0.0348 (10)	−0.0020 (7)	0.0184 (8)	−0.0001 (7)
C15	0.0505 (11)	0.0200 (8)	0.0386 (10)	0.0051 (7)	0.0253 (9)	0.0085 (7)
C16	0.0402 (10)	0.0290 (9)	0.0283 (9)	0.0129 (8)	0.0154 (8)	0.0106 (7)
C17	0.0284 (9)	0.0268 (8)	0.0207 (8)	0.0066 (7)	0.0119 (7)	0.0026 (6)
C18	0.0251 (9)	0.0401 (10)	0.0205 (8)	0.0085 (7)	0.0058 (7)	0.0056 (7)
C19	0.0177 (8)	0.0394 (9)	0.0206 (8)	−0.0018 (7)	0.0042 (7)	−0.0033 (7)
C20	0.0213 (8)	0.0230 (8)	0.0201 (8)	−0.0009 (6)	0.0092 (6)	−0.0015 (6)
C21	0.0251 (9)	0.0191 (8)	0.0465 (11)	−0.0031 (6)	0.0103 (8)	−0.0062 (7)
C22	0.0234 (8)	0.0166 (7)	0.0339 (9)	−0.0039 (6)	0.0113 (7)	−0.0057 (6)
C23	0.0343 (10)	0.0269 (8)	0.0359 (10)	−0.0052 (7)	0.0199 (8)	−0.0038 (7)
C24	0.0378 (10)	0.0290 (9)	0.0346 (10)	−0.0013 (8)	0.0067 (8)	0.0018 (8)
C25	0.0254 (9)	0.0303 (9)	0.0520 (12)	0.0013 (7)	0.0094 (9)	−0.0099 (8)
C26	0.0347 (10)	0.0425 (11)	0.0517 (12)	−0.0045 (8)	0.0274 (10)	−0.0111 (9)
C27	0.0376 (10)	0.0350 (10)	0.0339 (10)	−0.0020 (8)	0.0184 (8)	0.0005 (8)
N1	0.0208 (7)	0.0167 (6)	0.0358 (8)	−0.0038 (5)	0.0125 (6)	−0.0014 (5)
O1	0.0190 (5)	0.0248 (5)	0.0214 (6)	−0.0063 (4)	0.0068 (5)	−0.0028 (4)
O2	0.0223 (6)	0.0207 (5)	0.0254 (6)	−0.0069 (4)	0.0089 (5)	−0.0045 (4)
O3	0.0214 (6)	0.0200 (5)	0.0367 (7)	−0.0016 (4)	0.0114 (5)	0.0020 (5)
P1	0.0170 (2)	0.01769 (19)	0.0248 (2)	−0.00387 (14)	0.00742 (16)	−0.00084 (15)

### Geometric parameters (Å, °)

**Table:**

C1—C10	1.373 (2)	C16—C17	1.422 (2)
C1—C2	1.403 (2)	C16—H16	0.9300
C1—O1	1.4043 (18)	C17—C18	1.415 (2)
C2—C3	1.363 (2)	C18—C19	1.359 (2)
C2—H2	0.9300	C18—H18	0.9300
C3—C4	1.409 (3)	C19—C20	1.399 (2)
C3—H3	0.9300	C19—H19	0.9300
C4—C5	1.419 (2)	C20—O2	1.4039 (18)
C4—C9	1.422 (2)	C21—N1	1.4778 (19)
C5—C6	1.355 (3)	C21—C22	1.507 (2)
C5—H5	0.9300	C21—H21A	0.9700
C6—C7	1.404 (3)	C21—H21B	0.9700
C6—H6	0.9300	C22—C27	1.382 (2)
C7—C8	1.362 (2)	C22—C23	1.386 (2)
C7—H7	0.9300	C23—C24	1.386 (3)
C8—C9	1.415 (2)	C23—H23	0.9300
C8—H8	0.9300	C24—C25	1.373 (3)
C9—C10	1.430 (2)	C24—H24	0.9300
C10—C11	1.485 (2)	C25—C26	1.379 (3)
C11—C20	1.370 (2)	C25—H25	0.9300
C11—C12	1.439 (2)	C26—C27	1.378 (3)
C12—C13	1.418 (2)	C26—H26	0.9300
C12—C17	1.421 (2)	C27—H27	0.9300
C13—C14	1.370 (2)	N1—P1	1.6078 (14)
C13—H13	0.9300	N1—H1N	0.90 (2)
C14—C15	1.399 (2)	O1—P1	1.5921 (11)
C14—H14	0.9300	O2—P1	1.5992 (11)
C15—C16	1.356 (3)	O3—P1	1.4625 (11)
C15—H15	0.9300		
			
C10—C1—C2	123.23 (14)	C18—C17—C12	119.77 (14)
C10—C1—O1	120.16 (13)	C18—C17—C16	121.16 (15)
C2—C1—O1	116.56 (14)	C12—C17—C16	119.05 (15)
C3—C2—C1	118.71 (16)	C19—C18—C17	120.69 (15)
C3—C2—H2	120.6	C19—C18—H18	119.7
C1—C2—H2	120.6	C17—C18—H18	119.7
C2—C3—C4	121.42 (15)	C18—C19—C20	119.13 (15)
C2—C3—H3	119.3	C18—C19—H19	120.4
C4—C3—H3	119.3	C20—C19—H19	120.4
C3—C4—C5	122.35 (16)	C11—C20—C19	123.71 (14)
C3—C4—C9	118.95 (14)	C11—C20—O2	118.75 (13)
C5—C4—C9	118.69 (16)	C19—C20—O2	117.53 (13)
C6—C5—C4	121.42 (17)	N1—C21—C22	112.09 (13)
C6—C5—H5	119.3	N1—C21—H21A	109.2
C4—C5—H5	119.3	C22—C21—H21A	109.2
C5—C6—C7	119.85 (16)	N1—C21—H21B	109.2
C5—C6—H6	120.1	C22—C21—H21B	109.2
C7—C6—H6	120.1	H21A—C21—H21B	107.9
C8—C7—C6	120.63 (18)	C27—C22—C23	118.23 (16)
C8—C7—H7	119.7	C27—C22—C21	120.26 (16)
C6—C7—H7	119.7	C23—C22—C21	121.47 (15)
C7—C8—C9	121.15 (17)	C22—C23—C24	120.85 (16)
C7—C8—H8	119.4	C22—C23—H23	119.6
C9—C8—H8	119.4	C24—C23—H23	119.6
C8—C9—C4	118.19 (14)	C25—C24—C23	119.98 (18)
C8—C9—C10	122.24 (14)	C25—C24—H24	120.0
C4—C9—C10	119.56 (14)	C23—C24—H24	120.0
C1—C10—C9	117.58 (14)	C24—C25—C26	119.73 (17)
C1—C10—C11	120.47 (13)	C24—C25—H25	120.1
C9—C10—C11	121.94 (13)	C26—C25—H25	120.1
C20—C11—C12	117.54 (14)	C27—C26—C25	120.07 (17)
C20—C11—C10	120.06 (13)	C27—C26—H26	120.0
C12—C11—C10	122.26 (13)	C25—C26—H26	120.0
C13—C12—C17	118.00 (14)	C26—C27—C22	121.12 (18)
C13—C12—C11	122.95 (14)	C26—C27—H27	119.4
C17—C12—C11	119.01 (14)	C22—C27—H27	119.4
C14—C13—C12	121.03 (15)	C21—N1—P1	124.76 (11)
C14—C13—H13	119.5	C21—N1—H1N	117.2 (13)
C12—C13—H13	119.5	P1—N1—H1N	113.9 (13)
C13—C14—C15	120.62 (16)	C1—O1—P1	121.24 (9)
C13—C14—H14	119.7	C20—O2—P1	118.31 (9)
C15—C14—H14	119.7	O3—P1—O1	118.28 (6)
C16—C15—C14	120.15 (15)	O3—P1—O2	107.13 (6)
C16—C15—H15	119.9	O1—P1—O2	102.82 (6)
C14—C15—H15	119.9	O3—P1—N1	114.72 (7)
C15—C16—C17	121.13 (16)	O1—P1—N1	102.49 (7)
C15—C16—H16	119.4	O2—P1—N1	110.75 (7)
C17—C16—H16	119.4		
			
C10—C1—C2—C3	−2.0 (2)	C11—C12—C17—C18	−1.4 (2)
O1—C1—C2—C3	−179.17 (13)	C13—C12—C17—C16	−0.6 (2)
C1—C2—C3—C4	−3.5 (2)	C11—C12—C17—C16	177.21 (14)
C2—C3—C4—C5	−176.09 (15)	C15—C16—C17—C18	178.83 (16)
C2—C3—C4—C9	2.9 (2)	C15—C16—C17—C12	0.3 (2)
C3—C4—C5—C6	177.34 (16)	C12—C17—C18—C19	2.9 (2)
C9—C4—C5—C6	−1.7 (2)	C16—C17—C18—C19	−175.69 (15)
C4—C5—C6—C7	−0.7 (3)	C17—C18—C19—C20	−0.9 (2)
C5—C6—C7—C8	1.4 (3)	C12—C11—C20—C19	4.2 (2)
C6—C7—C8—C9	0.3 (3)	C10—C11—C20—C19	179.94 (14)
C7—C8—C9—C4	−2.7 (2)	C12—C11—C20—O2	−176.58 (12)
C7—C8—C9—C10	178.63 (15)	C10—C11—C20—O2	−0.8 (2)
C3—C4—C9—C8	−175.75 (14)	C18—C19—C20—C11	−2.8 (2)
C5—C4—C9—C8	3.3 (2)	C18—C19—C20—O2	177.96 (14)
C3—C4—C9—C10	3.0 (2)	N1—C21—C22—C27	82.3 (2)
C5—C4—C9—C10	−177.98 (14)	N1—C21—C22—C23	−100.01 (18)
C2—C1—C10—C9	7.7 (2)	C27—C22—C23—C24	1.4 (2)
O1—C1—C10—C9	−175.23 (12)	C21—C22—C23—C24	−176.33 (15)
C2—C1—C10—C11	−172.53 (14)	C22—C23—C24—C25	−0.4 (3)
O1—C1—C10—C11	4.6 (2)	C23—C24—C25—C26	−1.0 (3)
C8—C9—C10—C1	170.66 (14)	C24—C25—C26—C27	1.4 (3)
C4—C9—C10—C1	−8.0 (2)	C25—C26—C27—C22	−0.4 (3)
C8—C9—C10—C11	−9.1 (2)	C23—C22—C27—C26	−1.0 (3)
C4—C9—C10—C11	172.19 (13)	C21—C22—C27—C26	176.76 (16)
C1—C10—C11—C20	−51.9 (2)	C22—C21—N1—P1	159.02 (12)
C9—C10—C11—C20	127.90 (15)	C10—C1—O1—P1	68.19 (16)
C1—C10—C11—C12	123.65 (16)	C2—C1—O1—P1	−114.53 (13)
C9—C10—C11—C12	−56.6 (2)	C11—C20—O2—P1	76.15 (16)
C20—C11—C12—C13	175.66 (14)	C19—C20—O2—P1	−104.58 (14)
C10—C11—C12—C13	0.0 (2)	C1—O1—P1—O3	76.30 (12)
C20—C11—C12—C17	−2.0 (2)	C1—O1—P1—O2	−41.44 (12)
C10—C11—C12—C17	−177.66 (14)	C1—O1—P1—N1	−156.43 (11)
C17—C12—C13—C14	0.0 (2)	C20—O2—P1—O3	−173.99 (10)
C11—C12—C13—C14	−177.67 (15)	C20—O2—P1—O1	−48.64 (11)
C12—C13—C14—C15	0.8 (2)	C20—O2—P1—N1	60.22 (12)
C13—C14—C15—C16	−1.2 (3)	C21—N1—P1—O3	154.28 (14)
C14—C15—C16—C17	0.6 (3)	C21—N1—P1—O1	24.77 (16)
C13—C12—C17—C18	−179.16 (14)	C21—N1—P1—O2	−84.31 (15)

### Hydrogen-bond geometry (Å, °)

**Table:**

*D*—H···*A*	*D*—H	H···*A*	*D*···*A*	*D*—H···*A*
N1—H1N···O3^i^	0.90 (2)	2.01 (2)	2.9015 (17)	170.2 (18)

Symmetry codes: (i) −*x*, −*y*+1, −*z*+1.
